# Remarks on the Gibraltar Epidemic

**Published:** 1831-01-01

**Authors:** 


### REMARKS ON THE GIBRALTAR EPIDEMIC. By *David Barry*, M.D. Physician to the Forces.

OUR readers are aware that, in No. 26, of this Journal, pp. 337—350, we gave the substance of a paper on the Gibraltar Epidemic transmitted to us by Mr. Fraser, surgeon of the Civil Hospital of that fortress. In publishing that paper, we merely abbreviated some portions, where we thought them capable of abbreviation, and quoted the others *verbatim et literatim*, with the exception of omitting or softening down a *few words* conveying strong personalities, but without altering a single passage or the sense of it from beginning to end. Of the truth of this we shall leave Dr. Barry himself to judge—for to him we instantly transmitted the original document, as soon as his paper in reply appeared. We told Dr. Barry the conditions on which we inserted Mr. Fraser's paper, and that we would be willing to publish his reply on the same terms. Sometime afterwards we received a kind of rescript from Dr. Barry, stating that, unless we inserted the whole of his reply, verbatim et literatim, it should not appear in our Journal. This seemed somewhat abrupt, and we hesitated not a moment to inform Dr. Barry that we would make no engagement to publish any paper before we saw it—and that, at all events, we would not publish his paper unless he allowed us the same discretion of softening down terms, or abbreviating unimportant details, as we had done with Mr. Fraser's paper. This, of course, was declined by Dr. Barry; and then appeared the following document which we shall now re-publish *entire*, of our own accord—but which we, perhaps, should not have published *entire*, had Dr. Barry confided his paper to our care.

"*To the Editor of the London Medical and Physical Journal.*

SIR—The accompanying letter, as it now stands, addressed to Dr. JAMES JOHNSON, was originally intended to appear in his Journal, in reply to an article edited by him, in his last Fasciculus, and headed, 'Review of the Facts and Arguments brought forward by Dr. Barry, at the Royal College of Physicians, relative to the late Epidemic Fever in the Fortress of Gibraltar; by HUGH FRASER, Esq. Surgeon to the Civil Hospital, and late Assistant Surgeon 12th Regiment:' but as the Doctor insisted on being allowed to break up my letter into the shape of a review, and as I was naturally anxious that, if forced into a controversy, my arguments (such as they are) should be brought into action, in close column, unmutilated and undiluted by my adversary, I have thrown myself on your mercy, and now beg a place in your valuable and impartial pages.

There is no assertion contained in my letter that is not supported by reference to original, authentic documents, and, therefore, even those of your readers who may not have had an opportunity of making themselves acquainted with Mr. Fraser's paper will, I trust, find my observations worth their perusal, as tending to elucidate the history of the late Gibraltar epidemic.

I have the honour to be, Sir,
Your most obedient and obliged servant,
D. BARRY, M.D.
Physician to the Forces.

*Welbeck street; Sept.* 25*th*, 1830.

*Letter to* Dr. JAMES JOHNSON, &c.

SIR—As you have not told us where Mr. Fraser's original paper may be referred to, I take it for granted that what you have given of it is merely a hash, sliced from the raw, unpublished material, and served up to your readers, 'with a little of my own sauce,' to give it an 'eclectic' flavor. It is to be hoped, however, that Mr. F. will not suffer his lucubrations to remain in this mangled state, and that he will bring them out himself, ere long, in all their native richness. *En attendant*, I am glad to perceive that you appear to know your man. You seem to consider him as a sort of Perkins' steam gun, sent in advance of the main body by the leaders of his party, to mow down all preliminary obstacles, by being made to pour forth an interminable stream of offensive missiles, whilst you, acting 'condenser' to the machine, as you very facetiously designate yourself, intersperse the muddy torrent of abuse with occasional little puffs of thin, editorial vapour, 'few and far between.' But I am trifling with your reviewing dignity, and shall now proceed to business; first assuring you, that nothing, not even the proof afforded by your last fasciculus, that your pages may be sometimes made the vehicle of personal acrimony, shall induce me to descend to the use of that vulgar weapon.*

* The plainest answer which we can give to the above good-natured effusion, is Dr. Barry's own letter received while this sheet was passing through the press.

1. Your friend Mr. F. begins where his conscience must have told him he would probably find it convenient to leave off, viz. by an apology. He commences the fight under cover of a rather gauche paulo-post-futurum-kind of amende, thus : 'I shall be sorry if.' Tell him it is well ; but remind him, at the same time, that when my paper, which he has so furiously attacked, was read at the College of Physicians in London, he was in Gibraltar ; that it is still in possession of the College, and still unpublished ; that he, most certainly, has never seen it, and that, therefore, he must not pretend to know any thing whatever of its arguments, and only so much of its facts as may be collected from the little volauvent, à la periscope, which you were kind enough to toss up, at the time, from a memorandum given to you in an unlucky hour, by myself, and from memory. You now convert my simple facts into most learned postulates, (an honour for which I never designed them,) and in this form serve them up a second time. But 'facts are stubborn things,' as you and your protegé may find out hereafter.

2. Mr. Fraser tells you and your readers, that 'before the arrival of Dr. Pym in the garrison, in November, even the word contagion had become nearly obsolete,' &c. In reply to this, take the following letter, from the principal medical officer to his Excellency the Lieutenant Governor, through his military secretary. It is one of many similar documents of that period, still fortunately existing.

'*Gibraltar* ; 24*th Sept*. 1828, 9 *o'clock. Immediate. No*. 869.

'Sir : Nothing has yet been done about the infected bedding at the Naval Hospital. Pray allow me to order it over the line wall at Camp bay instantly, as much loss of life may be the consequence. The barrack master's plan, of taking it direct to the sandpit, is fraught with danger. It should be steeped in the sea for sixty hours at least before it goes to any place, where it may be mixed with other beds.

(Signed) 'J. HENNEN, M.D.'

'To Lieutenant-Colonel Marshall, Military Secretary.'

Can you, or your friend, quote any authority, living or dead, which affords stronger evidence of a full and perfect conviction of the reality of contagion, than the above? Pray compare this letter with those, by the same author, of the 29th August immediately preceding, since become the Alcoran of the unbelieving localist.

3. When Mr. F. shall have redeemed his promise of, 'more of this hereafter,' relatively to a paper, which he states I 'drew up,' shortly after my arrival in Gibraltar, to prove the noncontagious nature and local origin of the epidemic, I shall reply ; not before. I dare him to the proof.

4. 'The late epidemic (says Dr. Barry,) was a fever of one paroxysm.' My words in the original paper were, 'This disease, reduced to its most elementary form of expression, consisted of one, and only one, febrile paroxysm, terminating, from the second to the sixth day, either in a rapid and permanent return to health, or in the precursory, but apyrectic signs of speedy dissolution, black vomit, &c.' Your friend pithily pronounces 'the definition given,' (and that without having seen or heard it,) to be 'based in ignorance or error.' In the next paragraph he gives his own definition, thus : 'It

---

26, *Welbeck Street*, 8*th Nov*. 1830.

Sir—I have just seen the manuscript of Mr. Fraser's paper and hasten to convey to you this my unsolicited, and unqualified acknowledgment, that you have given the paper, in your Journal, with the most perfect fairness and fidelity ; and that in leaving out some parts, and in emending a few words, you appear to have been actuated by a wish to soften down the asperities of the original, without altering its purport.

Should any expressions of mine seem to indicate that I felt a different impression when I wrote my reply, I hereby retract them.

Your report of my paper, read before the Royal College of Physicians, in March last, I have already adopted, by stating that it was composed from a memorandum of my own, drawn up by myself, from memory.

Should you think this communication worth inserting in your next Fasciculus, it is much at your service.

I remain, Sir,

Your obedient servant,

D. BARRY, M.D.

Physician to the Forces.

*To Dr. James Johnson,*
*Physician to the King,*
*&c. &c. &c.*

N.B. Conscious of our own integrity on this point, we transmitted the original document to Dr. Barry merely for his satisfaction. The above explanation is that of a liberal and generous mind, and is all that is necessary to free us from any suspicion of unfair dealing.—*Ed.*

was a fever of a *continued form*, in which, in the cases of recovery, *the most perfect remissions took place.*' Now, sir, disdaining all attempts at sophism, of which I am accused, I would ask, can a fever exhibit 'the most perfect remissions,' and, at the same time, be 'of a continued form? If we do not attach a conventional meaning to words, they must cease to be intelligible symbols. Need I refer you to CULLEN's account of remittent fever? Need I say, that the word *paroxysm*, as used by that celebrated nosologist, comprehends the whole drama of fever, the three acts, cold, hot, sweating; and that a perfect *remission* is the apyrectic interval between two of these complete dramas; whilst *exacerbation* means only a higher colouring of the middle act, when, as in continued fever, this act is long enough to admit of its shades being observed. Allow your friend to go on, and, depend upon it, he'll try to persuade you, that a figure of a circular form can exhibit the most perfect angles.'

I shall not stoop to notice the intention to deceive, which your polished protegé appears capable of attaching to my definition, if it can be called one. He will not, surely, attribute this unworthy motive to his friend and colleague, Mr. PETER WILSON, in the view which he takes of the type of this fever, 'as it prevailed in Gibraltar in the Autumn of 1828,' in his Historical Sketch, published in the Lancet, No. 354, vol. ii. p. 424. These are his words: 'A fact resulting from my own experience in Gibraltar, namely, that the fever under consideration, which every year appears there, and which, in the Autumn of 1828, assumed the character of an epidemic, is as much a continued fever as our home typhus is. *As it exhibits, therefore, none of these remissions*, nor, even at a low temperature, ever breaks off into ague, it can be assigned no closer affinity to the family of intermittents, than results from the circumstance of its remote exciting cause being a product of the earth, or, in other words, not an emanation from the living body.' When it is recollected that Mr. P. Wilson was one of the medical officers of the civil hospital, where he resided during the whole of the epidemic; that he not only saw as many cases of the disease as Mr. F., but that he saw the very same cases; that he was the very originator and founder of the drain and privy pathology, and the Coryphæus of the non-contagion and local-origin sect, in Gibraltar; that, therefore, he is one of the last men in the world, who could be suspected of a wish to help me out of a scrape. When, I say, all these considerations are taken into account, his evidence must be deemed decisive of the nosological part of the controversy. Your friend's slip-slop descriptions, his 'lulls,' and 'commencements of a primary or temporary morbid association,' will probably induce you to part company from him on this point, and let him down *easy* in one of your next editorial parachutes. By the way, Mr. Wilson's clear, precise language on this subject will serve you for three or four very nice postulates.

5. Next comes a whole bundle of these things (postulates) which I am charged with having perpetrated, as to the cleanliness of the town of Gibraltar,* its municipal improvements, the comfortable condition of its poor, the moderate number of its population, &c. In order to beat down all these assertions at once, and to establish localism upon their ruins, Mr. F. gives, at full length, two official letters, [how did he procure the copies ?] from Dr. Hennen to his Excellency the Lieutenant Governor. On these letters I shall merely observe that, when Dr. Hennen wrote them, he was chief medical officer of the quarantine department, for which, independently of his military pay, he received one pound sterling per diem; that the ship Dygden had been admitted to pratique, *with his sanction*, about three weeks before;† that a rumor had now gained considerable credit in the garrison, that she had introduced the fever, then breaking out; that it could not be pleasant to Dr. H. to have it believed that an enemy had entered the fortress through the very pass for the guarding of which he was so liberally paid. But an enemy had actually made his appearance in the very centre of the citadel: who could he be? from whence had he come? The two letters answer these two questions. The first letter christens the stranger by the mystifying, pompous name of '*bilious, autumnal, remittent fever*,' with the word '*yellow*' occasionally appended.

* Dr. O'HALLORAN, in his interesting work on the Yellow Fever of the South and East Coasts of Spain, published in 1823, thus expresses himself, (p. 183,) 'General Don, when he assumed the command of that station, improved its condition so much that Gibraltar, from its being one of the most filthy towns in Europe, is *now one of the cleanest any where to be seen.*'

† In the face of information from the Board of Health, and British consul of the province of Valencia, dated 22d July, 'that this ship was infected with the yellow fever,' and from the Spanish consul of Gibraltar, who stated officially, on the 25th July, 'that a bad fever raged at the Havannah, by the latest accounts from that place.'

This, as far as a name could go, was calculated to give him the air of a native scorpion. The second letter decides his birth and parentage, found in the drains and privies of district No. 24, by Mr. P. Wilson, who, to do him justice, never acknowledged the fitness of the name. This letter, as you perceive, was penned at nine o'clock P. M., when, from the exertions of the day, the heat of the season, the pressure of great responsibility, the reports that had been spread, &c., the author's mind might not have been in that cool, unruffled state which the gravity of the occasion demanded. Indeed, the letter carries with it internal evidence of the justice of this remark. Read the three measures recommended to his Excellency's especial attention, as the *only means* of preventing a repetition of the horrors of 1813 and 1814.' viz.

1st. To sweep away certain sheds, lest they might become hot-houses of contagion.
2d. To appoint a committee to inspect these sheds and other unauthorized buildings.
3d. To order walking exercise for the police sergeants.

This mode of putting the case looks, I confess, rather ludicrous; but if you turn to the letter itself, you will find that I am fully borne out by its contents.

I shall reserve what further remarks I may have to make on these letters for a future occasion.

6. Surely your northern friend, Mr. Fraser, must be *daft*, or else there must be an error of the press, when he is made to say that 'the board of commissioners, the majority, at least, came to the conclusion that there was not the slightest proof for referring the introduction of our epidemic to the ship Dygden, or to any other vessel.' You shall soon see how the majority stood. There were *seven* members only on the Board. Mr. F. gives the opinions of *two* of these members, against importation, and in favor of local origin. To save space and time, you will, I presume, allow Dr. Pym and myself to be stanch importers. Of the three remaining opinions, you may judge for yourself: they are as follows:

COLONEL FALLA.

'I am of opinion that the late epidemic was not of local origin, but, from the strong presumptive evidence before the Board, that it was imported.'

(Signed) 'D. FALLA.'

'*Gibraltar*; 30*th April*, 1829.'

WILLIAM SWEETLAND, ESQ.

'After the most attentive consideration of the evidence which has been brought forward, I have discovered nothing which has carried conviction to my mind as to the cause or origin of the late epidemic fever. On the one hand, it has not been shown that any of the causes stated in support of the doctrine of its being of domestic origin, existed in a higher degree in the year 1828 than in many preceding years, when the garrison was free from that disease. On the other hand, no vessels arrived, during the last summer, having the yellow fever on board; nor has that disease discovered itself amongst any of the shipping in the port. In the absence, therefore, of any proof, on either side, I must decline hazarding any opinion on a subject which has baffled the researches of the most learned physicians of all countries, and continues to do so.'

(Signed) 'WILLIAM SWEETLAND.'

'*Gibraltar*; 30*th April*, 1829.'

DR. BROADFOOT.

'From every thing in my power to learn on the subject investigated by the Board, I cannot believe that the late epidemic originated here; but, on the contrary, I think it was imported.'

(Signed) 'ALEX. BROADFOOT.'

Thus you perceive that, of the seven members, four 'came to the conclusion' that the disease was imported; two, that it was not; whilst one declined coming to any conclusion. You will find out more clearly, by and by, with whom you have embarked your controversial fortunes.

*Mother Fani's Affidavit*. In order to comprehend this, you must refer to the proceedings of the Anglo-French commission, where you will find a declaration, signed by all its members, M. Chervin amongst the rest, dated February 1829, setting forth that this good lady showed such a palpable determination to mislead *(tromper)* the commission, that her evidence could not be admitted amongst their documents. Thanks to the liberality of the French government, these papers are now printed, and may, I hope, shortly be seen by the public. In these papers, and in the proceedings of the British Board of Commissioners, there is some curious evidence, tending to prove that the stink from drains and privies, on which you seem to lay so much stress, acted rather as a preventive against the fever, than as a cause of it, in Gibraltar in 1828.* By the way, I hope the College of Physicians will induce our government to print, *pro bono*, the documents connected with this epidemic. How it would abridge the badgering which I shall have to undergo from all the crazy endemic philosophers, who will now avail themselves of your '*condensing*' powers, which you are candid enough to confess you exercise, 'for the good of Mr. Fraser and the public.'

With regard to the nature of the disease of which the Fani children died,† you may consult the obituary register of the Roman Catholic church of Gibraltar, in which the word *febre* is opposite each of their names; also a certificate of the same, signed by the vicar, and laid before the Board of British Commissioners; the evidence of the man who tied the legs of one of them together, and placed the body, yellow with livid spots, in its coffin; the evidence of the woman who saw the other vomit black, and who declared that the child told her she had been on board ship the Sunday before; *enfin*, the posthumous evidence of the Doctor, Braulio Lopez, who attended them in their last illness.‡ Is Mr. F. soft enough to imagine that the relict of two smugglers would confess to him and his *confrères*, but, above all, to Mr. Shea, the government lawyer, that her family ever had any illicit intercourse with ships in quarantine? Which do you think would be most likely to deal in complicated 'made-up falsehoods, a boy not quite thirteen years of age, and a few washerwomen,' or a hardened veteran smuggler? Such as the latter, however, (to use his own expression,) 'is the front and bearing of one channel, on which Mr. F. rests his proofs.'

As a judge of cases, do you not think that the case of *strangulated hernia* of old Fani is *well* made out? *pain in the throat*,|| '*no vomiting*.' The treatment, too: no attempt at taxis, no bleeding, no operation, though he had been seen by Mr. F. and others.

8. I have now arrived at the case of Mr. Fraser's servant-maid. Her name, he will recollect, was Maria, 'a Portuguese; she had lived with him three years.' There can be no doubt as to her identity. He says that this woman was attacked 'on the *third* of August, 1828, by a fever, which he considered at the time, in common with Mr. Peter Wilson, late of this (the civil) hospital, to be one of those sporadical attacks of yellow fever, which every now and then appear there.' Mark this well, I entreat you! Mr. F. *now* solemnly asserts that this woman was attacked by yellow fever 'on the *third*:' I say that she was attacked by this disease on the *twenty-third* of August. Let this disputed point be the touchstone of our respective claims to honest precision in facts and dates, and therefore to public confidence and respect. As to the '*obliquity*' with which he charges me on this subject, I shall fling it indignantly back upon him, with such overwhelming conviction of misstatement, on his part, that no ingenuity can ever extricate him from it. Should the dark spot which you allowed your protegé to attempt upon my probity be now fixed upon his own, he has himself to blame. But, after all, there is so much of vagueness, of the irritation of disappointment and party rancour about his whole paper, so much of the appearance of being pushed on by a coterie, that his *slight* anachronism, of *twenty days*, may be accounted for, without resorting to his own ungenerous imputation.

1*st Evidence*. The Commission of Origin and Cause, of which I had the honour of being a member, and secretary, aware of the great importance of *first cases* in the history of all epidemics, and anxious to obtain correct information as to the dates and circumstances of the earliest attacks of fever in 1828, resolved, at their first sitting in Gibraltar, on the 28th January, 1829, (4th resolution,) 'That all medical practitioners

---

* It is recorded by VILALBA, the historian of Spanish Epidemics, that, in the great plague of London, the physicians ordered all drains, privies, and other sources of stench to be opened, and fully exposed, for the purpose of checking the contagion. They attributed much success to this measure.

† The first cases of fever that occured in Gibraltar in the autumn of 1828. 'Yet, from the woman's own account (Mrs. Fani) little or no doubt can be entertained that it was *the fever*.'—WILSON's Historical Sketch, *Lancet*, No. 352, p. 326.

‡ Extract: 'Some days before Dr. Braulio Lopez got sick, I was informed by him that the two children of Felix Fennic, or Fani, named Salvador and Catalina Fennic, had died of the black vomit in district No. 24. The former died on the 17th August, the latter on the 20th of the same month.'
(Signed) 'JOHN CORTEZ, Surgeon.'
*Gibraltar; 30th January*, 1829.
'To Dr. Barry, Secretary to the Board, &c.'

|| See PYM on Pain in the Throat, as accompanying Bulam, p. 228-9.

within this garrison, civil as well as military, be officially called upon to furnish to the Board, by Friday next, a circumstantial return of the first case of disease which each of them may have treated, or which may have come to their knowledge, in this garrison, *last year*, and which they may have considered, then or since, *identical with the fever which has so lately prevailed here in an epidemic form*.' As it had been asserted by some, that sporadic cases of yellow fever appear annually in Gibraltar, care was taken to word this resolution so as to preclude all quibbling about the precise day when the disease assumed an epidemic form; and by taking in the whole of the year 1828, to prevent the possibility of having *a new first case* introduced, at any future period, under the pretext of its being looked upon as a sporadic, at the time.

A circular letter was immediately drawn up, containing the words of the resolution. It was dated that very day, signed by me, as secretary to the commission, and sent by an official messenger to every medical man in the garrison. The following is a true copy of Mr. Hugh Fraser's return to this circular.

'Sir: In reply to your letter of the 28th instant, I beg to inform you, that the first case of the epidemic fever, which came under my observation, took place in a child of Mr. Martin's, residing in No. 24 district, on the *eighteenth* August, 1828. The history of this family is so generally known, that I deem it unnecessary to say more. Should the Board of commissioners require any further information from me, I shall be most happy to give it.'

(Signed) 'HUGH FRASER.'

'*Civil Hospital*; 30*th January*, 1829.

'To Dr. Barry, secretary to the Board, &c.'

This letter requires no comment when compared with Mr. F.'s present statement, 'that his own servant-maid was attacked by yellow fever, on the *third* of August.'

2*d Evidence*. The morning states drawn up by Mr. Tucker, then chief clerk in the principal medical officer's office, now deputy purveyor. Mr. Tucker then attended the civil hospital in a medical capacity, and knew this woman. He noted her, with Dr. Hennen, on the 2d of September, as convalescent from fever, with this remark, in his own hand-writing: 'Servant to Mr. Fraser, who has been ill more than a week; treated in his own house.' She is again described in the morning state of the 3d of September: 'Mr. Fraser's servant-woman.' These two returns are the second and third of the kind made out that year: copies of both are now before me, authenticated by Mr. Tucker. Here I must beg to observe, that I have never met with more integrity, more correctness, nor more striking impartiality, than in this gentleman. To him is due, much of what has been left of accurate record, of that fatal scourge of 1828.

3*d Evidence*. The following will be found in Mr. P. Wilson's Historical Sketch, already quoted from the Lancet, No. 353, vol. ii. p. 388: 'But a servant-maid of the surgeon of the hospital became, towards the close of August, affected with fever, when only the case of the 21st of August had been admitted, and though she lived in the division of the hospital most remote from the ward where that patient lay, and was never known to have gone near him, it was artfully attempted to hook in her case, to form a link in the chain of contagious influence.'

I shall say nothing of the evidence of this woman's sister, who was employed in the hospital at the time, and in the age of whose infant this attack marked a monthly epoch; the girl's own declaration to those about her sick bed, 'that she had caught the *plaga*,' requesting her mistress not to come near her, &c.

Finding no account of this case in the books of the civil hospital, to which I had unlimited access, as principal medical officer of the garrison, during a part of 1829, I asked Mr. F. if he had taken notes of it? to which he replied, 'not at the time, but he had since, from memory.' Would he be kind enough to let me see them? 'No: but (added he,) you had better take no notice of it.'

Mr. Fraser may well say 'that it is not in ignorance this case is brought forward:' but let him now gather the fruits of his unworthy aspersion; let him suffer the recoil of the foul missile which he aimed at me; let him emerge, if he can, from the inevitable conclusion that he either deceived the Board of Commissioners in 1829, or the public in 1830.

9. Mr. Fraser, all through his paper, evidently piques himself upon using strong language, and indeed, (to give him his due,) it is sometimes so strong, that it covers his meaning with an impenetrable shield, from behind which, this literary Ajax fancies that he deals most murderous knocks. Can you guess what he means by 'moral testimony being accepted to confirm the question of liability, or nonliability?' Confirming a question, I should say, means, bothering an already doubtful point so effectually as to render it doubly questionable. This you will see hereafter is exactly what he intended.

The Board of Nonliability, which Mr. F., with his usual accuracy, says was composed of Drs. Chervin, Louis, Trousseau, Dr. Barry, and myself, besides some half dozen Spaniards,' counted amongst its members, in addition to the above, Dr. Broadfoot, principal medical officer, Mr. Dow, acting deputy inspector of hospitals, Mr. Dix, staff surgeon, and Mr. Thurston, civil practitioner.

Mr. Fraser proposed that every indisposition, during an epidemic, should be taken as a case of the prevailing disease, and that '*moral evidence*' should be considered sufficient to decide upon the reality of any alleged case of double attack. These propositions were supported by Mr. Chervin, who had a long list of cases drawn up, on this plan. The Spanish physicians endeavoured to explain, *moral evidence*, to each other, by translating it into the word, *tradicion*. M. Louis, the president, though a very clear-headed man, could scarcely comprehend it, as applied to pathology. Mr. F., for the benefit of his British friends, illustrated the matter, something in this way: John comes before the Board, and states, or Mr. F. states for him, that his brother, James, who is supposed to be in South America, had been indisposed with headach, in Gibraltar, in October, 1828, and that their mother, who is dead, had declared that both her sons had been also sick, in the Autumn of 1813. Here is a case of second attack. Upon this sort of evidence being refused, not by *vivâ voce* vote, but by ballot, Messrs. Chervin and Fraser became *dissentient*; the other *eleven members* signed the report. What is the College of Physicians about? Why does it not influence the Government to print this report, to which the high character of M. Louis, as a pathologist, and the importance of the question itself, give a value which few professional documents possess?

10. I am now quite sick of Mr. Fraser's endless postulates. 'No hospital servant was taken ill at all for about one month after the commencement of the epidemic, *though in the closest attendance upon the sick.*' This is the very reason why they had not been attacked, previously to the introduction of John Holdroyd, of the 12th regiment, amongst them, on the 2d September. But, as one swallow makes no Summer, let us say the epidemic commenced amongst the troops on the 4th September, which is one day before Mr. Fraser and his colleagues knew that any thing extraordinary had occurred to disturb the public health. They had not yet seen the cordon, nor received the printed bann issued, at two o'clock on the morning of the 5th September, by the Spanish physicians just returned to Algesiras, from Gibraltar. In this circular, they repeat to their British confréres of that garrison, the hints which had already been given to them, in vain, by the old women of the rock, viz. that they had the *vomito negro* amongst them.

I have now before me official returns signed by the surgeons of corps, proving that no less than fifty-nine hospital orderlies were seized with the epidemic between the 5th September and the 10th October; viz.

| | |
|---|---|
| Royal Ordnance | 12 |
| 12th regiment | 7 |
| 23d regiment | 3 |
| 42d regiment | 9 |
| 43d Light Infantry | 13 |
| 73d regiment* | 1 |
| 94th regiment | 14 |
| | 59 |

When we have learnt, in a manner that precludes all possibility of doubt, that these fifty-nine seizures far exceed the whole of the attacked, during the period mentioned, amidst a civil population, of more than two thousand, inhabiting the south district, where the military hospitals were all placed, and where the first civil case occurred, on the 5th September, in the person of Acres, the brother of Holdroyd's reputed wife;†

* This regiment had its hospital on Windmill hill.—Dr. Barry begs it may be stated, that from a memorandum which he has since found amongst his Gibraltar papers, he has reason to believe, that the hospital of the 73d Regiment was not established on Windmill Hill, until after the period alluded to in the note.

† This young man, a day labourer, lodged with his sister and her pretended husband, in district that there were only 164 soldier-orderlies employed about the military sick, from the beginning to the end of the epidemic, and perhaps not half that number at any given time; how shall we account for this immense disproportion of attacks between the civil and military, in the first month, without leaning a *leetle* to contagion? I forgot to say, that not one word of these *postulates*, about the military orderlies, belongs to Mr. F.: the whole has been put into his *cat's paw*, to pluck my explanation out of the fire: you will allow, I think, that he has burnt his fingers.

I have some hopes of bringing *you* round to the true faith; but as to your friend, I despair of bringing him even to reason. He has plunged into the arena, or rather has been let loose into it, with such blind and reckless fury, that there is no managing him. He puts one in mind of a fierce Andalusian bull, just rushing into light, from his narrow stall, butting even at his own shadow. 'Cornu qui jam petit, et pedibus qui spargit arenam.'

11. Thank my stars! I am now arrived near the conclusion: I have reached Mr. F.'s very becoming remarks on the treatment of the disease, and the comparative success of the different medical people then in the garrison. But the idea of bulls having crossed my imagination, I cannot resist the opportunity of noticing one of the many pasquinades, to which the epidemic gave rise, and which were privately circulated, in Gibraltar, during my residence there. It was in the Spanish language, and, in my opinion, afforded evidence that all the wit of Spain was not buried with Cervantes. This pasquinade was in the shape of a handbill, announcing a grand bull-fight, in the '*Placa de Toros* of Gibraltar, by permission of His Excellency the Governor, &c.' It set forth that several first-rate bulls, of the true *black breed* called 'la Epidemia,' would be killed by the most celebrated *matadors*.* The cattle were said to have been brought in by the well-known herdsmen.—The medical men of the garrison were designated as the bull-fighters, each having a *nom de guerre*, or Spanish *sobriquet*, indicative of some prominent peculiarity of character, or conduct. Some of these were highly amusing, and did honour to the native wit of the remote Calpe. I cannot now recollect who appeared, in colossal characters, as *first*, and *second matadors*, *first*, and *second spadas*,† *picadors*, *banderillas*, &c. but, I dare say, some of Mr. F.'s friends, who understand Spanish, have shewn him this little *jeu d'esprit*, and explained its allusions.

Now, as to my humble share in this exhibition. I arrived in the bay of Gibraltar on the 8th November, landed on the 10th, observed the general localities of the place, saw and conversed with most of the civil practitioners, made myself acquainted with the different modes of practice, civil as well as military, and their results; on the 14th, I addressed a letter to the principal medical officer, of which the following is an extract.

'Let me entreat you to press upon His Excellency, when you see him this day, the crying necessity of adopting, without the loss of a single hour, such measures as will prevent any more sick from being sent to the naval hospital. The accumulation of infectious* disease there, has been, in my mind, the leading cause of the great excess of the proportion of military, over civil deaths. You can execute no plan in so short a time, that will so effectually, and so rapidly check the frightful mortality of the troops, and at the same time, so largely increase the confidence of the garrison in yourself. Give me the authority and the thing shall be done this day.

(Signed) 'D. BARRY, M. D.
Physician to the Forces.'

'To Dr. Broadfoot, P. M. O.'

* I use the word infectious, as synonymous with contagious.

His Excellency granted the desired authority, without a moment's delay, and that very evening, the 12th, 42d, and 43d regiments began to send their fresh cases to some sheds close to the neutral ground, on which they were then encamped. From that day to the close of the epidemic, not a man was sent by any of these corps, to the naval hospital. The sheds-hospital was placed under my immediate superintendence.

---

No. 24. Finding himself ill, about the beginning of September, he went to his mother's in the south, who, with a Portuguese family, occupied a cottage close to, or under the bridge of the naval hospital. Here he remained concealed for two or three days, but was discovered on the 5th, and sent to the civil hospital. The whole of the Portuguese family were successively taken ill, some days after. These were the first civil cases in the south. (Vide the documents of the Anglo-French commission, and of the British commission.

* *Matador*: he who despatches the bull, with a sword, after the animal has been sufficiently tortured.

† *Spada*, or swordsman; *Picador*, lanceman; *Banderilla*, tormentor or flagbearer.

The annexed is a brief statement of the admissions, recoveries, and deaths in this little establishment, together with a general outline of the treatment pursued, from its formation on the 14th November, to the 14th December following, taken from official returns, now in my possession, drawn up at the time, and signed by the medical officers in charge of the respective corps.

| | Admitted. | Convalescent. | Died. |
|---|---|---|---|
| 12th regiment | 22 | 18 | 4 |
| 42d regiment | 11 | 8 | 1 |
| 43d Light Infantry | 23 | 17 | 4 |
| Sappers | 2 | 2 | 0 |
| Totals | 58 | 45 | 9 |

At the close of the returns, four were not yet considered convalescent. Seven only are returned, whose convalescence was delayed beyond the seventh day from the attack.

Of the nine deaths, there are only two so late as the sixth day of the disease.

Of the fifty-eight admitted during the month, eleven had mercury administered to them, either before their admission, or after, and without my approbation. Of these eleven, five died. Twenty-eight were bled from the arm, and two from the temporal artery; in no case to more than sixteen ounces. Of the thirty thus treated, three died: in one of these three, there was profuse hemorrhage from the temporal artery in the night. This practice may not have been quite so vigorous as that of Mr. F. and his supporters; but I have the satisfaction of reflecting, that the loss of life fell very far short of fifty-three and a half per cent. upon the wole of the sick admitted.

12. With regard to the knowledge of the epidemic, be it great or small, which I may have acquired at Gibraltar, those who knew me there will, I believe, allow, that no man worked harder to make himself acquainted with its characters and its history than I did, during a residence of fourteen months. The documents of the three commissions, of which I had the honour of being a member, will, I trust, bear me out in this assertion. But it is evident that Mr. F. and those who have furnished him with the dignified observations (for they are not his own,) which he makes in this portion of his paper, are determined to establish, *per fas, aut nefas*, a monopoly for themselves in this disease, more particularly in its anatomical characters. I shall not deny that their post-mortem facilities were most abundant; much more so than those of their colleagues. So jealous, indeed, are they on this latter head, that they will not allow me the melancholy honour of having been in at more than 'eight or ten deaths.' But even this is more than I can claim, having declined to participate in the treatment of two of the fatal cases. Pray remind these gentlemen, in your usual classic manner, that the pompous, but empty Mussulman, who has plunged his ruthless mattock oftenest, and deepest, into the debris of the Parthenon, in the destruction of which he has anticipated the scythe of time, is not to be considered on that account the only judge of what had been its architectural, and sculptured ornaments; that he who daily delves in the quarry, or the mine, is not always the most enlightened geologist. As to myself, I was placed, on my arrival, as you have seen, in charge of the sick of three, out of the seven regiments, of which the garrison consisted. I witnessed autopsies, at the civil hospital, with Dr. Pym and others; at the military hospital, with my colleagues of the Anglo-French commission. At my own hospital I missed none, and all the fatal cases were opened there. I took notes on the spot, of the living and the dead. I examined registers of dissections, where they had been kept. During the last eight months of my residence on the rock, not a single fatal case occurred, either in the civil, or military hospitals which I did not see examined after death, and of which I did not take notes, at the time. But why descend to talk of these matters? A few cases, diligently observed, and carefully noted at the moment, are worth a cartload of confused recollections, however mysteriously puffed off as the fruits of exclusive research. So much for your friend's defiance of 'contradiction' to his borrowed slander.

13. Knowing that *you* are fond of postulates and other '*sesquipidalia*,' I shall give you two or three on the subject of mercury, as a naval and military medicine.

1st. It should never be given, in either service, when the disease can be cured without it, because it renders the constitution, once seriously affected by it, much less capable than it had been of resisting the influence of cold and wet for years after, and therefore often leads to the invaliding, if not the death, of many an able seaman and good soldier.

2d. There was not even one case, in the whole of the late epidemic of Gibraltar, which recovered under the use of mercury, that would not have recovered more quickly, more comfortably to the patient, and much more profitably to the service, without a single grain of it.

3d. Almost the only cases of tedious convalescence we had at Gibraltar, and I saw the whole of them with Dr. Pym, were men who had used mercury in large quantities. The fever itself hardly ever, I might say never, left organic disease behind it.

Thanks to our director general, he has done much to discourage the unnecessary exhibition of this drug, in those diseases to which young soldiers are particularly liable.

14. The motives which induced me, in consultation with my colleagues of the sheds hospital, first to limit the use of this mineral to its mildest combinations with a vegetable aperient, and afterwards to suspend its administration in toto, were of the most irresistible kind. Such motives had, perhaps, never occurred before, and probably never will again; but as your forlorn hope is *hors de combat*, I shall reserve my fire for the main body.

Should you think that any of your friend's assertions still rest upon even the semblance of defensible ground, be kind enough to let me know. You shall have facts and documents until you cry 'Hold, enough!' In the mean time, presuming that *your engine must halt to take in a fresh supply of fuel*,

I remain, sir, your obedient servant,

D. BARRY, M.D.

Physician to the Forces.

P.S. By way of a set-off against your friend's poetical flourish about 'the thousand sick of some fever on board the Havannah ship, who all died without proving any thing,' (which I forgot to notice sooner,) you shall have my official opinion on the subjects of local origin and importation of the Gibraltar fever, as it stands attached to the proceedings of the Board of Commissioners.

DR. BARRY.

'I am of opinion that the fever which prevailed in an epidemic form in this town and territory, last autumn, was not of local origin, but that it was imported. I have been induced to form the above opinion from the following reasons:

'1st. Because I consider that it has been satisfactorily proved to the Board that this fever is identical with the yellow fever, or black-vomit fever of the West Indies, with the epidemic fever which, at different periods within the last thirty years, has committed such ravages in this garrison, and in other parts of the south of Spain: that it is essentially different from every fever indigenous in any part of Europe, in its mode of attack, its symptoms, its duration, its anatomical characters, and, above all, in its affecting the same individual but once during life.

'2dly. Because I conceive that, after the most minute examination of all the medical and other evidence which could be procured, it has not been satisfactorily shown to the Board that even one case of fever has occurred here, within the thirteen years immediately preceding 1828, identical with the late epidemic fever, excepting four which occurred in the year 1817, in the persons of lighterman and Jews, who had been in the habit of having intercourse with West India ships; although it is presumable that, during that period, all the physical causes inherent in this territory, and capable of generating disease, had been in full action, and although the population was more dense and the houses less commodious during these years than in 1828, as stated to the Board by the best informed and most respectable evidences.

'3dly. Because I conceive that there is no evidence before the Board to show that there is any source of malaria within this territory, and because I conceive that it has been fully proved to the Board that the effluvia arising from drains, even when they have been most offensive, had no share in producing the late epidemic.

'I am of opinion that the late epidemic disease was imported, and afterwards propagated here.

'1st. Because I conceive that there is ample evidence before the Board to prove that this disease is capable of being propagated either by direct communication, or by means of the clothes of persons who had been affected by it.

'2dly. Because I consider it proved to the Board, by the clearest evidence, oral as well as documentary, that several of the ships which arrived here last summer and autumn, from yellow-fever countries, had suffered much from sickness and deaths on their passage; amongst others, the Swedish ship Dygden: that four or five of this ship's crew died in the Havannah, and two on her passage here, of yellow fever; nine having been sick during the passage; that the mate of the ship was ill about the 24th July, whilst in quarantine, and was not reported to the inspector of health; that, on the 7th August, the day after the ship had been admitted to pratique, one of her crew was received into the civil hospital here, as a case of 'intermittent fever from the Havannah;' that one of the men shipped on board this vessel here, to assist in navigating her to Cadiz, was taken ill a few days after having gone on board of her, and continued so for five or six days; that mattresses and other effects were thrown overboard from this ship whilst she lay in quarantine here and in Cadiz; that the clothes of the men that died on board this ship were sold to sailors who landed from her, about the 6th August last; that the sister of a sailor who landed from this ship, and who had had the black vomit fever in the Havannah, immediately before he embarked from that place for Gibraltar, received a bag of clothes belonging to that sailor, and fell sick on the 20th August; that the health guard, Francis Teste, who was placed on board this ship on the 27th July, declared to several persons that she brought the yellow fever to this garrison; that the sister of this health guard, who, on the 11th August, assisted to wash his clothes which he brought from on board this ship, fell sick, according to his own declaration to the Board, on the 21st August; that the very first persons who were attacked by the late epidemic were connexions of sailors and health guards, young persons who had been recently on board-ship, and washerwomen.

'3dly. Because, at the moment that the sailors of the Dygden landed here with the clothes of the men who had died on board her of yellow fever, all the collateral circumstances favorable to the propagation of disease were present, viz. the sultry calm of a southern autumn; the peculiarly sheltered and unagitated state of the atmosphere of Gibraltar; the steady, high range of the thermometer; the abundance of subjects fitted, from their age and freshness, to receive the contagion, and from their never having undergone the protecting influence of a former attack of this singular and dreadful malady; and, lastly, because the ship Dygden was admitted to pratique on the 6th August, and because the very first case of the epidemic occurred on the 12th of the same month.

(Signed) 'D. BARRY, M.D.
'*Gibraltar; April* 30, 1829.' Secretary to the Board.'"

Of the stormy discussion which, we have reason to know, is approaching, we shall take occasional analytical notice, without identifying ourselves with either party. Meanwhile, we have no hesitation in saying that Dr. Barry's observations and official documents demand a full and deliberate reply from Mr. Fraser, and the gentlemen on his side of the question. Dr. Barry's best friends will probably regret the *jocular* manner in which he has treated a subject, that is little adapted to such a style of writing.—*Ed.*

---

